# Public Health Messaging and Measures During COVID-19: The Experiences of Family Caregivers

**DOI:** 10.1093/geroni/igab046.2106

**Published:** 2021-12-17

**Authors:** Deirdre McCaughey, Kristin Flemons, Whitney Hindmarch, Gwen McGhan

**Affiliations:** University of Calgary, Calgary, Alberta, Canada

## Abstract

To mitigate the effects of COVID-19, Health Ministries across Canada have enacted numerous public health measures. Our mixed methods study examined the effect of COVID-19 related public health messaging and measures for family caregivers (FCGs) of people living with dementia (PLWD). Of the 230 FCGs completing the survey, most frequently used information sources were television, family/friends, and websites. FCGs over 60 more often used television, newspaper and radio versus websites and social media. FCGs rated public health messaging as good-excellent (64%) especially messaging around the disease spread, symptoms, and finding information. 46% believe the restrictions in long-term care facilities went beyond necessary with 97% reporting restrictions have negatively impacted them. 84% were willing to undertake personal protective equipment and infection control training to ensure continued access to PLWD. Focus groups highlighted concerns about continued access to PLWD, quality of care provision, and increased social isolation’s impact on dementia progression.

